# Experimental Investigation of Relationship between Humidity Conditions and Degradation of Key Specifications of 0.1–1.2 GHz PA in 0.18 μm CMOS

**DOI:** 10.3390/mi13081162

**Published:** 2022-07-22

**Authors:** Shaohua Zhou, Cheng Yang, Jian Wang

**Affiliations:** 1 School of Microelectronics, Tianjin University, Tianjin 300072, China; zhoushaohua@tju.edu.cn (S.Z.); ych2041@tju.edu.cn (C.Y.); 2 Qingdao Institute for Ocean Technology, Tianjin University, Qingdao 266200, China

**Keywords:** class-A, CMOS, humidity, performance degradation, power amplifier (PA)

## Abstract

The specification of power amplifiers (PA) is closely related to humidity variation, and few reports on the humidity properties of PA are available in the literature. Therefore, an experimental study of PA specifications was conducted under different humidity conditions to elucidate the relationship between the degradation of PA specifications and humidity conditions. This paper studies and provides results of the degradation of a PA subjected to different humidity levels. The experimental results show that the S_21_ and output power decrease with the increase in humidity. The main cause of this degradation is the decrease in oxide capacitance and increase in threshold voltage with increasing humidity, resulting in a reduction of transconductance and an increase in on-resistance. The results of this study can guide designers in designing compensation circuits to achieve some degree of compensation for the degradation of PA specifications.

## 1. Introduction

With the rapid expansion in the wireless industry [1,2], the power amplifiers (PAs), which play critical roles in developing wireless devices and establishing communication [3,4,5], are in high demand. For long-distance transmission, the antennas are driven mainly by the PAs [6,7,8]. The PAs employed in such applications are exposed to environmental conditions [9,10] and sometimes extreme working conditions such as rapid changes in temperature and humidity [11,12,13,14]. According to the literature, humidity caused 10% of equipment failures at US coastal bases [15]. The performance of the PA can be affected by the humidity conditions that may result in system failures [16,17]. A failure is an event that ends the life of a particular product [18,19]. However, we never use PA in existing communication systems until its end of life. Before reaching the end of a PA’s lifetime, that PA’s performance has already begun to degrade and cannot meet the required specifications of the designed systems [20,21].

From a systems point of view, the system cannot tolerate a drop in PA’s specification, such as gain, bandwidth, etc. [19]. Once the performance of the PA is degraded, even if the magnitude of performance degradation is very small, it can lead to catastrophic damage to the overall system [22,23,24]. This means that when the performance of the PA degrades below a predetermined threshold, even if the degradation is minor, the PA is incapable of meeting the design specification [20,21]. Therefore, figuring out the PA’s degradation concerning humidity variations becomes crucial to ensure the proper system operation. Unfortunately, the characteristics of gallium nitride (GaN)-based PAs in a humid environment have limited studies in the literature [20], while studies of Complementary Metal Oxide Semiconductor (CMOS)-based PAs exposed to varied humidity conditions are entirely lacking.

This paper uses a CMOS Class-A PA to systematically investigate the PA’s specifications degradation under different humidity levels from an experimental point of view. The PA’s specifications degrade as humidity rises. Furthermore, the PA’s degradation mechanism under high humidity conditions is elucidated. This paper aims to help PA designers consider the humidity effect of PA in advance in the design process.

## 2. Designed PA and Experimental Setup

**Figure 1:**
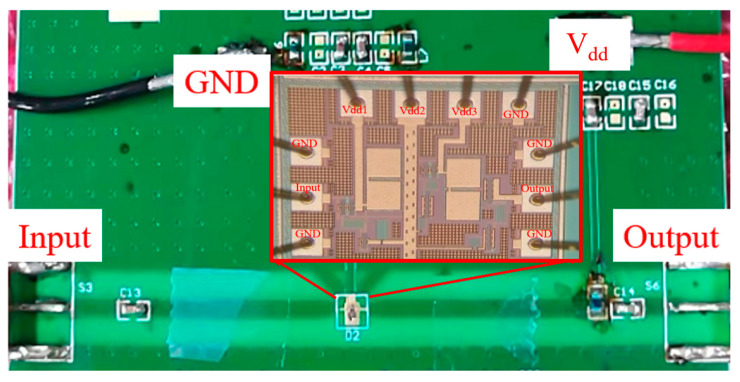
The chip microphotograph of the CMOS PA.

The 0.1–1.2 GHz CMOS broadband PA is a technological achievement result of the national science and technology project. The project requires the design of a broadband PA that can be used in industry-specific networks, and the study of the temperature and humidity characteristics of the PA is required to lay the foundation for future product finalization. Therefore, we conducted the humidity characteristics study with this PA in this context. The PA was processed in Global Foundry 1P6M 180 nm CMOS (The Foundry Company, Sunnyvale, CA, USA) with a chip area of 0.414 mm^2^ [25], as shown in Figure 1. As you can see from Figure 1, there are ten pads in the chip, five of which are ground pads, three are power pads, and two are input and output pads. The supply voltage of V_dd1_, V_dd2_, and V_dd3_ is 3.3 V. Special attention should be paid to the fact that the chip ground (GND) pad must be connected to the real earth; otherwise, it will cause the test to fail, or the test result will be abnormal.

**Figure 2:**
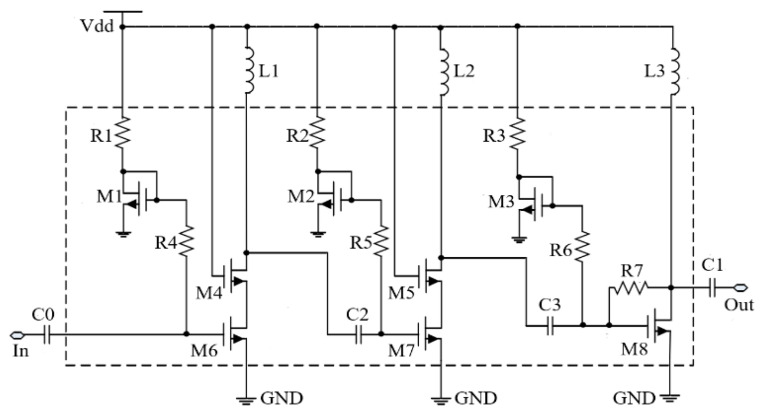
The schematic diagram of 0.1–1.2 GHz Class-A PA.

As shown in the circuit diagram of Figure 2, a PA with a three-stage single-ended structure was used for the experiments, and the first and second stages of the PA were cascaded to increase the gain of the PA. In the third stage, a common source amplifier + resistive negative feedback structure is used to achieve high power output. The PA uses a 3.3 V powered thick oxide N-channel metal-oxide semiconductor (NMOS) transistor in a 0.18 μm CMOS process to increase output power, with a minimum gate length of 0.35 μm.

**Figure 3:**
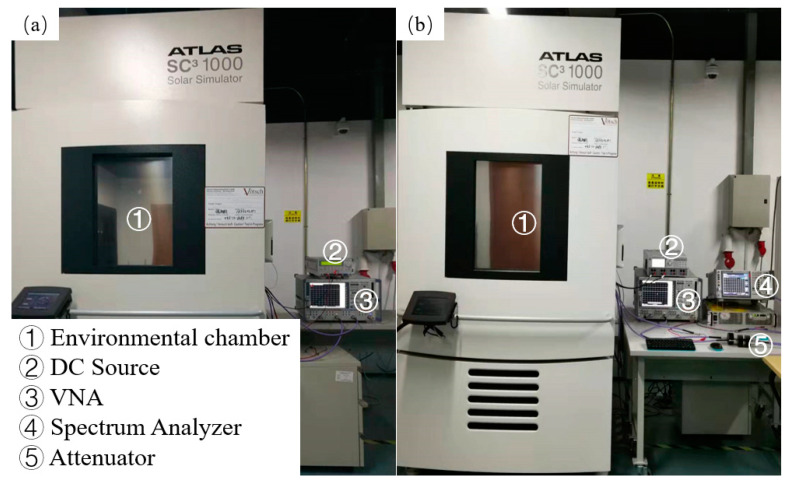
Measurement environment: (**a**) S-parameter; (**b**) output power.

This experiment must be performed in a Solar Climatic Test Cabinet (SC^3^ 1000 MHG) (Vötsch Industrietechnik Gmbh, Balingen, Germany) (Figure 3). Unfortunately, the existing on-chip test platform does not support humidity experiments. Therefore, the chip to be tested can only be packaged on a printed circuit board (PCB) board (as shown in Figure 1) and then placed in the environmental test chamber for measurement. The PCB board used in this experiment is Rogers 4350 (Rogers, Killingly, CT, USA). According to the datasheet of the Murata inductor and capacitor (Murata, Nagaokakyō, Japan) and Rogers 4350, the performance of the inductor, capacitor, and PCB can be considered constant. Therefore, the PA’s specifications degradation in this experiment can be mainly caused by the changes in the transistors in the PA. This way, the transistors’ measurement in a humidity-changing environment is achieved indirectly.

To study the humidity characteristics of individual transistors, existing on-chip test benches can also fail to provide a humidity measurement environment. Therefore, individual transistors are packaged on a PCB and measured in an environmental test chamber. However, the transistor’s input/output impedance is not the typical 50 Ω of the standard test system. Therefore, measuring a single transistor’s performance under humidity variation is impossible using the standard test environment. Therefore, this paper does not discuss the characteristics of transistors in different humidity environments by directly measuring them.

The R&S HMP4040 (R&S, Munich, Germany) was used to provide DC bias, an attenuator (Rosenberger, Tittmoning, Germany) was used to reduce the power at the spectrum analyzer’s input, and the R&S ZVA40 (R&S, Munich, Germany) and FSV30 (R&S, Munich, Germany) were used to record the experimental output results. Since the humidity range that the environmental test chamber can measure is 10% to 90% Relative Humidity (*RH*), this paper reports the variation of the S_21_ and output power in the humidity range of 10% to 90% *RH* only.

## 3. Results and Discussions

### 3.1. The S_21_

**Figure 4:**
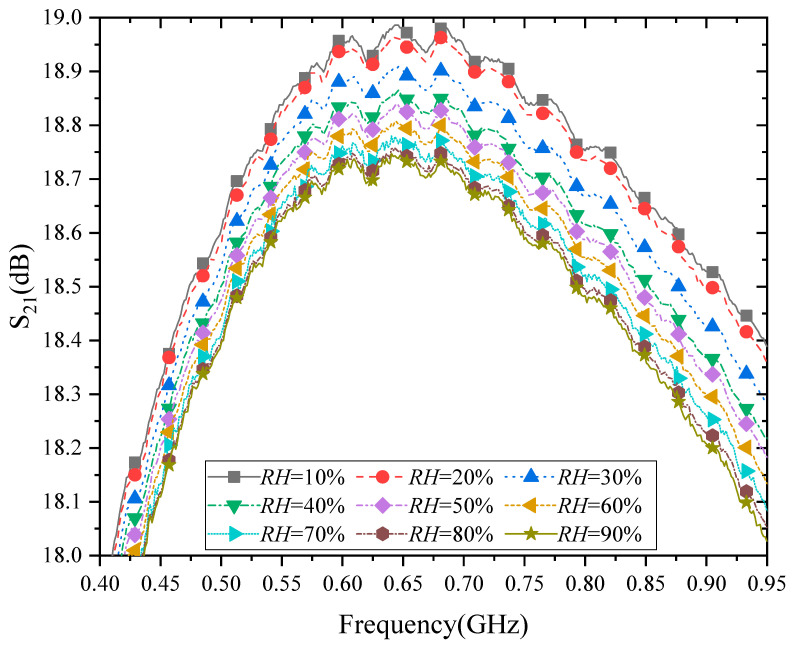
Measured small-signal gain with frequency variations.

The measured S_21_ is as in Figure 4. The measured S_21_ is not only the function of frequencies but also varies according to different humidity conditions. For example, at 500 MHz, the gain difference between 10% to 90% *RH* is 0.21 dB, but at 900 MHz, the difference is 0.32 dB. The differences in gain for the same humidity conditions will be different at different operating frequencies.

**Figure 5:**
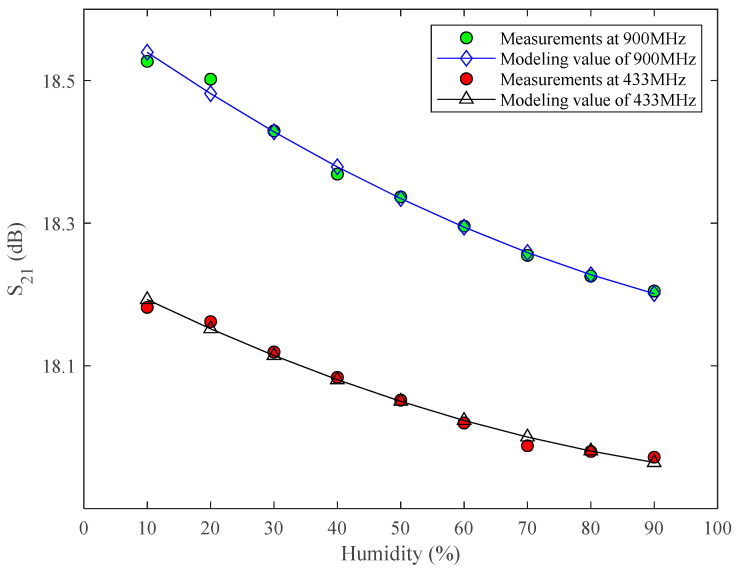
Measured S_21_ vs. humidity.

Figure 5 gives the details of the humidity dependence of S_21_. In this figure, we took 433 MHz and 900 MHz as examples based on two common communication methods: interphone and public mobile phone. If we take S_21_ at 20% *RH* as a typical value and 0.25 dB drop as an acceptable criterion, the maximum operating humidity is around 70% *RH*. This means that the gain of the power amplifier can meet the requirements below 70% *RH*. However, as shown in the introduction, if the S_21_ of the PA degrades is below 18.25 dB (e.g., the degradation is minor), the S_21_ of the PA will not be able to meet the demand at this time.

As shown in Figure 4 and Figure 5, S_21_ of PA decreases with the increase of humidity. What is the cause of the degradation of S_21_ of PA with humidity? Next, this paper will discuss and analyze it in detail. For Class A, the PA operates at bias points *V_gs-M8_* = 1.7 V, *V_ds-M8_* = 3.3 V, and *I_ds-M8_* = 60 mA. Therefore, the PA works in the saturation region.

In the saturation region, we obtain [26,27,28]:

(1) $$I_{ds}=\frac{W_{g}\mu_{n}C_{ox}}{2L_{g}}{\left(V_{gs}-V_{th}\right)}^{2}$$

where *W_g_* is the gate width, *μ_n_* is the carrier mobility, *C_ox_* is the gate oxide capacitance per unit area, *L*_g_ is the gate length, *V_gs_* is the gate voltage, *V_th_* is the threshold *V_ds_* is drain voltage.

The gate oxide capacitance per unit area is found to be [26,27,28]:

(2) $$C_{ox}=\frac{\varepsilon_{ox}}{t_{ox}}$$

where *ε_ox_* is the permittivity of the oxide, and *t_ox_* is the thickness of the oxide.

As reported in the literature [29,30], the accumulation of water diffusion through the polysilicon sidewalls at the oxide/silicon interface near the edge of the source/drain junction will increase the thickness of the oxide. Therefore, the drain current and gate oxide capacitance per unit area will decrease with increasing oxide thickness according to Equations (1) and (2).

In addition to the possible reasons mentioned above, another reason for the decrease of the drain current is the increase of the threshold voltage with the rise in the humidity. This experimental work’s finding aligns with the literature’s theoretical confirmation [31].

The transconductance of a MOSFET is defined as the change in drain current concerning the corresponding change in gate voltage [26,27,28]

(3) $$g_{m}=\frac{\partial I_{ds}}{\partial V_{gs}}$$

Therefore, according to Equation (3), the transconductance of the saturation region is [26,27,28]

(4) $$g_{ms}=\frac{W_{g}\mu_{n}C_{ox}}{L_{g}}(V_{gs}-V_{th})$$

It is already known from the previous discussion that the oxide capacitance (*C_ox_*) decreases, and the threshold voltage increases as humidity rises. Therefore, according to Equation (4), it is known that the transconductance will decrease with the increase of humidity. And the transconductance is generally considered to be S_21_. Therefore, it can be observed that S_21_ decreases with the rise in humidity.

In summary, there are two reasons for the degradation of S_21_. The first reason is the increase in threshold voltage with increasing humidity. The second reason is that *C_ox_* decreases with increasing humidity. It may be noted that these reasons were not observed directly by the experiment earlier.

In addition, we also fitted the relationship between S_21_ and humidity variation at 433 MHz and 900 MHz (as shown in Figure 5) based on the quadratic polynomial model, and the expressions are:

(5) $$S_{21–433\mathrm{MHz}}=1.7765\times 10^{-5}RH^{2}-4.6327\times 10^{-3}RH+18.2375$$

(6) $$S_{21–900\mathrm{MHz}}=2.2451\times 10^{-5}RH^{2}-6.4763\times 10^{-3}RH+18.6023$$

where *RH* is the humidity.

From Equations (5) and (6), the expressions of S_21_ with the temperature at 433 MHz and 900 MHz are formally the same, and both are quadratic functions of *RH*. Only the coefficients are different.

### 3.2. The PA’s Output Power

**Figure 6:**
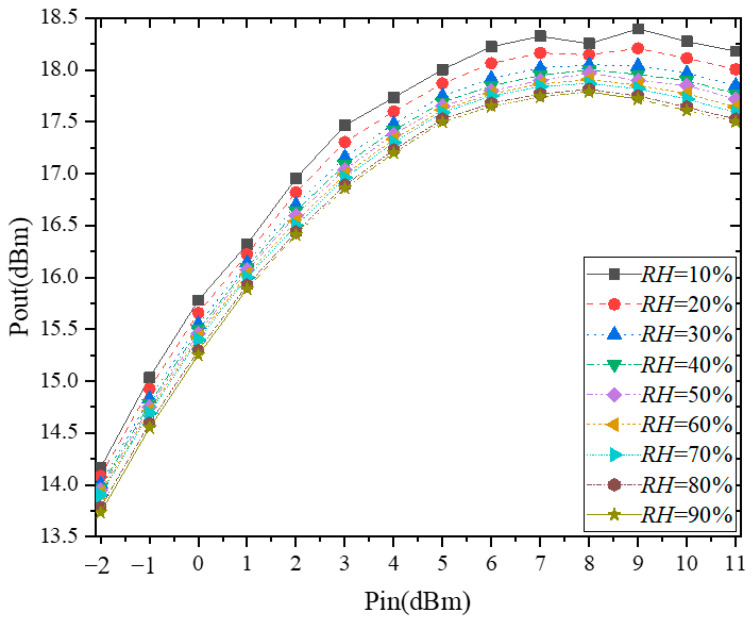
Measured output power vs. humidity at 433 MHz.

We studied the characteristics of the output power of the power amplifier at 433 MHz, as this is a typical radio frequency for different humidity levels. The humidity characteristics of the output power as shown in Figure 6. It can be seen from it that output power decrease with increasing humidity. And when the humidity changes the same, the magnitude of the degradation of output power at different input power is not the same. As discussed in sub-Section 3.1, it is already known that the decrease in *C_ox_* and the increase in threshold voltage cause the drain current to decrease with increasing humidity. Therefore, the following analysis focuses on the causes of the decrease in output power with increasing humidity.

In the following, we will discuss and analyze specifically the causes of the decrease in output power with increasing humidity. The on-resistance is [32]:

(7) $$R_{on}=R_{0}+\frac{kT^{\alpha }}{{\left|V_{gs}-V_{th}\right|}^{\beta }}$$

Here, *k* is a constant, and *R_0_* represents the initial value of the resistance. The coefficients *α* and *β* are 1.5 and 0.2, respectively.

According to Equation (7), the on-resistance will increase with an increasing threshold voltage. As known from the discussion in 3.1, it has been demonstrated experimentally and in the literature that the threshold voltage increases with increasing humidity. This means that the on-resistance will increase with increasing humidity. The results of our experimental analysis are the same as in the literature [31]. Therefore, an increase in the on-resistance will lead to a decrease in the output power.

**Figure 7:**
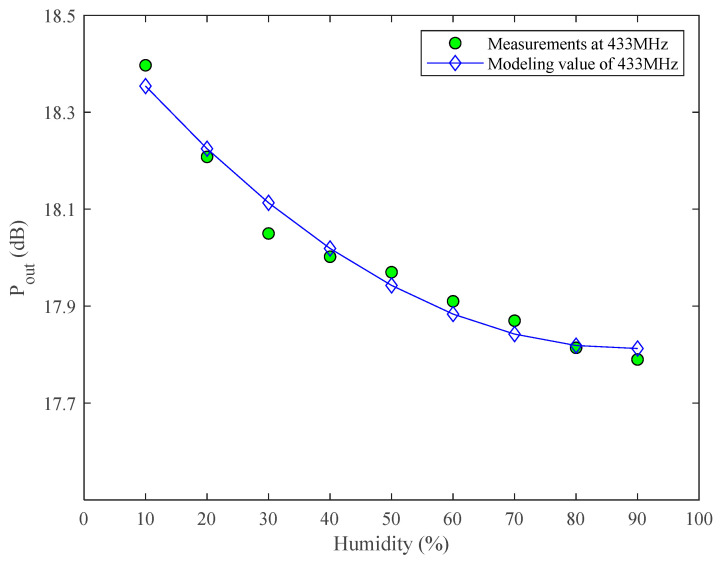
Saturation output power vs. humidity at 433 MHz.

Figure 7 gives the relationship between saturation output power and humidity at 433 MHz, from which the saturation output power decreases with the increase of humidity. At the same time, we fit the relationship of saturation output power with humidity based on the quadratic polynomial model, and the expression is:

(8) $$P_{out}\left(sat\right)=8.7944\times 10^{-5}RH^{2}-1.5564\times 10^{-2}RH+18.5010$$

From Equation (8), the saturation output power ($P_{out}\left(sat\right))$ with humidity is similar to the expression of S_21_ with humidity, and both are related to quadratic humidity. Only the specific coefficients are different.

Since we only measured the output power at 433 MHz during the previous measurements, the output power at 900 MHz is not provided.

### 3.3. The Power Added Efficiency

**Figure 8:**
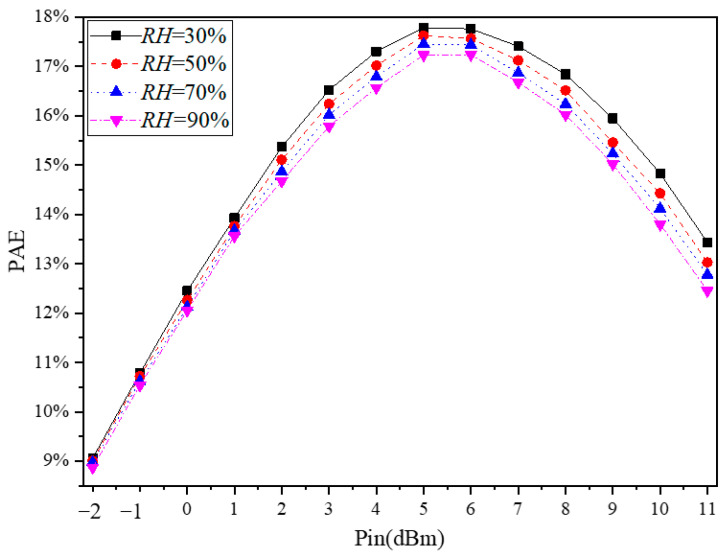
Measured PAE vs. humidity at 433 MHz.

The relationship between the power added efficiency (PAE) and humidity at 433 MHz is shown in Figure 8, from which PAE decreases with the increase of humidity. According to the definition of PAE, PAE is proportional to the output power; i.e., PAE decreases as output power decreases. The reason for the change of output power with the humidity has been discussed in sub-Section 3.2, so the reason for the reduction of PAE with the increase of humidity is the same as the reason for the decrease of output power with the rise in humidity.

**Figure 9:**
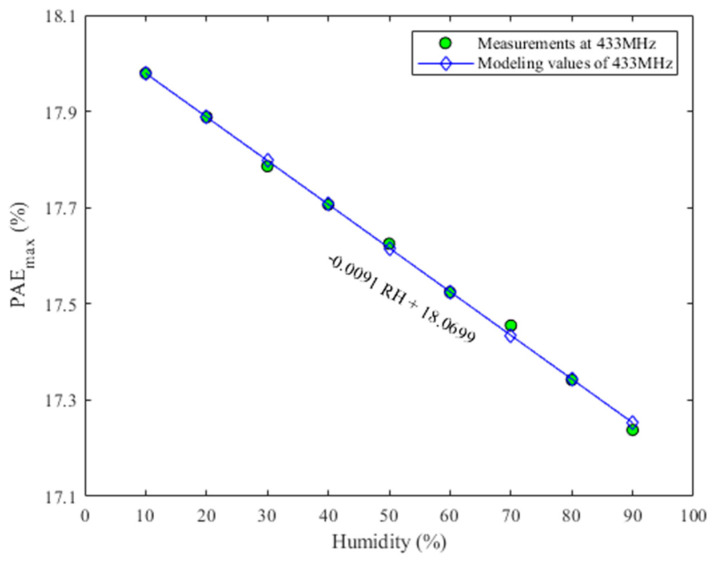
Measured maximum PAE vs. humidity at 433 MHz.

It should be noted that considering that the curves of S_21_ and output power changing with humidity in Figure 4 and Figure 6 are too dense, only PAE under four humidities are given in Figure 8 to observe the relationship between PAE and humidity change more clearly. Figure 9 shows the relationship between the maximum PAE and humidity at 433 MHz. At the same time, the relationship between the maximum PAE and humidity is fitted, and its expression is as follows

(9) $${PAE}_{max}=-9.0780\times 10^{-3}RH+18.0699$$

According to Equation (9), the maximum PAE is linear with the change of humidity, which is different from the relationship between S_21_ and saturated output power with humidity change.

## 4. Conclusions

This paper investigates PA specifications’ characteristics at different humidity levels from an experimental point of view. This paper study and provide results of the degradation by the humidity of only one PA. It is found that the S_21_ and output power degrades with the increase of humidity. The main reason for this degradation is that the *C_ox_* decreases, and the threshold voltage increases as humidity rises.

However, no common pattern for the degradation of the two specifications, S_21_, and the output power, was found during the study. This indicates that the specification degradation experiments must be continued at this stage for many PAs to extract sufficient experimental data. This will guide the extraction of common patterns of specification degradation for the prediction of specification degradation and compensation of specification degradation.

## Data Availability

Not applicable.
